# Expression Comparison of Oil Biosynthesis Genes in Oil Palm Mesocarp Tissue Using Custom Array

**DOI:** 10.3390/microarrays3040263

**Published:** 2014-11-13

**Authors:** Yick Ching Wong, Qi Bin Kwong, Heng Leng Lee, Chuang Kee Ong, Sean Mayes, Fook Tim Chew, David R. Appleton, Harikrishna Kulaveerasingam

**Affiliations:** 1 Sime Darby Technology Centre, Universiti Putra Malaysia, 1st Floor, Block B, UPM-MTDC Technology Centre III, Lebuh Silikon, 43400 Serdang, Selangor, Malaysia; E-Mails: kwong.qi.bin@simedarby.com (Q.B.K.); lee.heng.leng@simedarby.com (H.L.L.); david.ross.appleton@simedarby.com (D.R.A.); harikrishna.k@simedarby.com (H.K.); 2 European Bioinformatics Institute, Wellcome Trust Genome Campus, Hinxton, Cambridge CB10 1SD, UK; E-Mail: ckong@ebi.ac.uk; 3 School of Biosciences, University of Nottingham Malaysia Campus, Jalan Broga, 43500 Semenyih, Malaysia; E-Mail: sean.mayes@nottingham.ac.uk; 4 Department of Biological Sciences, Faculty of Science, National University of Singapore, Lower Kent Ridge Road, Singapore 117543, Singapore; E-Mail: dbscft@nus.edu.sg

**Keywords:** microarray, oil palm, mesocarp, gene expression

## Abstract

Gene expression changes that occur during mesocarp development are a major research focus in oil palm research due to the economic importance of this tissue and the relatively rapid increase in lipid content to very high levels at fruit ripeness. Here, we report the development of a transcriptome-based 105,000-probe oil palm mesocarp microarray. The expression of genes involved in fatty acid (FA) and triacylglycerol (TAG) assembly, along with the tricarboxylic acid cycle (TCA) and glycolysis pathway at 16 Weeks After Anthesis (WAA) exhibited significantly higher signals compared to those obtained from a cross-species hybridization to the *Arabidopsis* (*p*-value < 0.01), and rice (*p*-value < 0.01) arrays. The oil palm microarray data also showed comparable correlation of expression (r^2^ = 0.569, *p* < 0.01) throughout mesocarp development to transcriptome (RNA sequencing) data, and improved correlation over quantitative real-time PCR (qPCR) (r^2^ = 0.721, *p* < 0.01) of the same RNA samples. The results confirm the advantage of the custom microarray over commercially available arrays derived from model species. We demonstrate the utility of this custom microarray to gain a better understanding of gene expression patterns in the oil palm mesocarp that may lead to increasing future oil yield.

## 1. Introduction

Oil palm is one of the most productive oil crops with average oil yields of 4.2 tons of oil per hectare annually in Malaysia compared to other major world oil crops such as rapeseed and soybean with 1.2 and 0.4 tons per hectare, respectively [1,2]. Oil palm mesocarp tissue can accumulate up to 90% of its dry weight as oil at maturity, the highest known level of accumulation in the plant kingdom [3,4]. With the increase in global vegetable oil consumption, yield-related traits are the key target for plant breeders. Due to its economic importance, extensive research has focused on deciphering the underlying mechanisms and pathways influencing the efficient oil production machinery in the oil palm mesocarp tissue [3,4,5,6,7,8].

Recently, high throughput approaches, including transcriptome sequencing and microarray analysis, have been adopted to study trait-related pathways and provide an in-depth knowledge of the underlying mechanisms involved in oil production, drought, disease tolerance, and tissue development among others. These approaches have been adopted in both plants and animals [4,6,9,10,11,12,13,14,15]. Microarrays have been widely used due to their efficiency for analyzing global patterns of expression in a single experiment [16]. Currently, microarray analysis is still cheaper than comparative analysis methods using transcriptome sequencing (e.g., RNAseq), especially when large numbers of samples are being evaluated. However, commercially available and well annotated microarrays for plants are limited to several model and major crop species. Due to this limited availability, one approach has been to cross-hybridize RNA samples on microarrays from other plant species [17,18]. The success of cross-hybridization microarray experiments relies largely on the hybridization efficiency of the target genome to the cross-species probe sequences. The accuracy of gene expression measurements will also depend on the evolutionary distance between the target and the microarray design plant species, as well as the relative rate of evolution within the classes of genes under evaluation.

## 2. Experimental Section 

### 2.1. Experimental Setup and Sampling

A total of 16 *tenera* palms (*Elaeis guineensis* Jacq.; *dura* × *pisifera*) with similar genetic backgrounds (Serdang Avenue *dura* × AVROS *pisifera*) were selected from Sime Darby’s oil palm breeding program. These palms were selected from a total of more than 100 trees planted in the same field in a breeding trial located in East Estate, Carey Island, Malaysia, based on the homogeneity of vegetative and growth characteristics, but differentiated by different oil yields and bunch analysis traits that had been recorded over five years. No severe pest or disease issues were observed for any of the individuals during experimental period. Six different inflorescences of each of the 16 selected palms were open-pollinated over a two years period and harvested at 12, 14, 16, 18, 20 and 22 WAA, as described previously [19,20]. The full inflorescence was harvested and fresh oil palm fruits taken were randomized and selected for each time point. Fresh mesocarp tissue was separated from the randomized oil palm fruits immediately after the samples were taken in the field and frozen in liquid nitrogen before storing at −80 °C after transfer to the laboratory.

### 2.2. Oil Palm Mesocarp RNA Extraction

Total RNA was extracted from the oil palm mesocarp tissue using the following RNA extraction method. The mesocarp tissue was first ground to a fine powder in liquid nitrogen. The extraction buffer contained 0.1 M Tris-HCl, pH 7.6, 0.1 M NaCl, 6% p-aminosalicylic acid, 1% SDS, and 0.35% β-mercaptoethanol. The buffer was added to frozen ground material at the rate of 4 mL/g plant tissue, vortexed thorough and extracted with phenol/chloroform. An additional chloroform:isoamylalcohol cleanup step was performed after the phenol/chloroform extraction steps to improve the purity of the RNA. The supernatant was precipitated using 3 M LiCl followed by an ethanol precipitation. The pellet was redissolved in DEPC-treated distilled water and again precipitated with 3 M LiCl followed by another round of ethanol precipitation. The precipitate was finally dissolved in DEPC treated distilled water and stored at −70 °C.

The concentration and purity of total RNA was determined by spectrophotometric quantification (Thermo Scientific, Nanodrop ND-1000, Wilmington, DE, USA). The AU 260/280 and AU 260/230 were measured and samples with a ratio of 1.8–2.0 were utilized. Gel electrophoresis was also performed on 1 µg of total RNA using a 1% agarose gel in TAE buffer to further determine the integrity and quality of the RNA. Samples that passed the initial quality tests were further analyzed using an Agilent Bioanalyzer (Agilent, Bioanalyzer 2100, Santa Clara, CA, USA), allowing the ratio of the 28S to 18S peaks to be determined. Samples with 28S to 18S ratios of greater than 2:1 and an RIN (RNA Integrity Number) score greater than 7 (out of 10) were utilized for further work.

### 2.3. Building the Transcriptome, Custom Design of the Oil Palm Mesocarp Array and Agilent Commercial Array

A consensus transcriptome sequence was built from reads generated from sequencing oil palm mesocarp tissues collected from samples harvested at different WAA. The reads generated by Roche 454 GS-FLX sequencers were assembled using the Newbler program V2.5 (Roche 454 Life Sciences, Branford, CT, USA). After sequence assembly, sequences shorter than 100 base pairs, as well as those originating from organelles and rRNA, were removed, leaving 31,804 sequences. The sequences have been deposited in the European Nucleotide Archive (ENA) (accession number(s): LM611910–LM643713). In order to undertake a global analysis of the mesocarp gene expression, the transcripts were compared to the Gene Ontology database using BLAST2GO (version 2.3.5) [21]. This process classifies the genes according to the molecular function, biological process or cellular component. Biological pathway analysis of the transcripts was carried out by comparison with plant sequences annotated to reference pathways in the KEGG database [22]. For the processes above, the E-value cutoff for the BLASTx program was set to be 10^−5^.

The custom oil palm mesocarp array probes were designed based on these 31,804 sequences with the annotations obtained by comparing isotig sequences to the Uniprot database [23]. The custom gene expression oil palm mesocarp array was designed using the Agilent eArray web-based application in a 2 × 105K format. Probes were designed using the Agilent internal design program through the eArray website [24]. Each of unique transcriptome sequences was represented by three distinct probes. Agilent 60-mer SurePrint technology was used for array printing.

In the cross-species study, the 4 × 44K *Arabidopsis* (V4) Gene Expression Microarray (G2519F-021169) and the 4 × 44K rice gene expression microarray (G2519F-015241) from Agilent Technologies were used for hybridization with the RNA from the 16 WAA samples. The array consists of 43,803 probes for both *Arabidopsis thaliana* and rice. The Arabidopsis microarray was designed based on various databases including; NCBI Reference Sequence Database (RefSeq) (July 2008), UniGene (May 2008), TAIR 8 cDNA (April 2008), TIGR (June 2006), TIGR Plant Transcript Assemblies (June 2006) and ATHI (Jan 2004) [25]. The Rice microarray was designed based on resources from National Institute of Agrobiological Sciences, RefSeq and GenBank 2007 [25].

### 2.4. Synthesis of cRNA, Microarray Hybridization and Scanning

Total RNA samples from mesocarp were individually treated and labeled with a one-color (Cy3) dye according to the Low Input Quick Amp Labeling protocol (version 6.0; December 2009) provided by Agilent. A total of 100 ng of total RNA was used to synthesize cRNA labeled with Cy3 dye. Total RNA of 100 ng in 1.5 µL was mixed with 2 µL of Agilent One-Color Spike Mix. T7 promoter primer was added into the mixture and the reaction made up with Nuclease-free water to 5.3 µL. The reaction mixture was incubated at 65 °C for 10 min using a thermal cycler (BIO-RAD, C-1000, Hercules, CA, USA). After incubation, the reaction mixture was incubated on ice for 5 min. To reverse transcript the mRNAs to cDNA, a total of 4.7 µL cDNA Master mix was added to the previous reaction mixture. This consisted of 2 µL of 5X first strand buffer, 1 µL of 0.1 M DTT, 0.5 µL of 10mM dNTP mix and 1.2 µL of AffinityScript RNase Block mix. The reaction mixture was mixed thoroughly by pipetting up and down. After briefly centrifuging, the reaction mixture was incubated at 40 °C for 2 h, followed by 70 °C for 15 min using a thermal cycler (C1000, BIO-RAD). After the cDNA synthesis process, the reaction mixture was kept on ice for 5 min, before 6 µL of transcription mixture was added giving a total volume of 16 µL. This step was performed to transcribe cDNA to cRNA and incorporated the Cyanine 3-CTP dye to cRNA during the transcription process. The transcription mix consist of 3.2 µL of 5X transcription buffer, 0.6 µL of 0.1 M DTT, 1 µL of NTP mix, 0.21 µL of T7 RNA Polymerase Blend, 0.24 µL of Cyanine 3-CTP, made up with nuclease-free water to a volume of 6 µL. The reaction was mixed and incubated at 40 °C for 2 h using a thermal cycler. Labeled cRNAs were then purified using Qiagen’s RNeasy mini kit (Hilden, Germany) as recommended by Agilent. The quality and quantity were assessed using the Spectrophotometer ND-1000 (Thermo Scientific). A total of 1.65 µg of purified labeled cRNA was used for hybridization. The purified cRNA was mixed with 25 µL of 10X blocking Agent, 5 µL of 25X Fragmentation buffer and made up to a volume of 120 µL with nuclease-free water. The reaction was mixed and incubated at 60 °C for 30 min to fragment the cRNA and cooled on ice immediately after fragmentation. A total of 125 µL 2X GE Hybridization Buffer HI-RPM was mixed with the fragmented cRNA and hybridized onto the arrays at 65 °C for 16 h in a rotating hybridization oven. After hybridization, 2 steps of washing were performed with wash buffer 1 and 2 for 1 min each. The array was then air-dried for a few seconds before proceeding with image scanning using the Agilent microarray slide scanner (SG11350602). The slides were scanned using the green dye channel with scanning resolution of 5 µm at 20 bit of dynamic range. 

### 2.5. Data Extraction, Normalisation and Comparisons

Raw microarray data were extracted from scanned images by using the Feature Extraction software (version 10.7.31; Agilent) [26]. Background normalization was carried out using the normexp algorithm whereas normalization between the samples was carried out using quantile normalization [27,28]. Both normalization steps were implemented using the R package of limma (Linear Models for Microarray Data, version 2.13.1) [29]. The remaining parameters in limma were set to default value. For an expression difference to be determined as statistically significant, the log fold change must be ≥0.6 and false discovery rate (FDR) ≤ 0.05. Comparisons of selected candidates between oil palm mesocarp, rice and the Arabidopsis microarray were carried out based on the normalized signal intensities produced.

### 2.6. Quantitative Real-Time PCR

Validation of the microarray expression data was performed using quantitative real-time PCR (qPCR). The first strand cDNA prepared from pooled biological replicates of palms at each stage of maturation was used. Two micrograms of total RNA from each sample was used in a reverse transcription reaction using Omniscript Reverse Transcriptase with standard conditions as recommended by manufacturer (QIAGEN). The first strand cDNA synthesis was primed by random hexamer primers. Specific primers were then designed based on the in-house mesocarp transcript database at Sime Darby using the Primer Premier 5.0 software [30]. The qPCR reaction mix consists of 5 μL of a 5X dilution of cDNA, 0.8 μL of forward and reverse primers (10 mM), 10 μL of BIO-RAD iTaq^™^ Fast SYBR GREEN Supermix with ROX and topped up with distilled water to 20 μL. The PCR cycling conditions were based on optimized conditions suggested by BIO-RAD with 95 °C (1 min) for 1 cycle and followed by 95 °C (15 s) and 55 °C (35 s), for 40 cycles. Relative expression of each transcript was analyzed using qBase Plus 2.0 [31] and normalized against multiple reference genes. In this study, Cyp2 and GRAS were used as previously published [32,33].

## 3. Results and Discussion

### 3.1. Array Design and Sequence Annotation

Cross species microarray hybridization is a common approach used to study gene expression profiles of poorly annotated species [10,34,35]. Davey *et al.* [34] for example utilized the commercial *Arabidopsis* array (ATH-1 Affymetrix GeneChip®) and Rice array (Rice Affymetrix GeneChip®) to examine patterns of gene expression in banana under abiotic stress. They identified 2910 differentially expressed transcripts from *Musa* spp. in response to drought and found several transcripts that co‑localized to known rice QTLs that were previously identified through drought experiments. The cross species approach was applied due to the availability of a complete genome sequence and detailed publically-available resources. However, some reports caution that results obtained using cross species arrays may not reflect the true expression within the species under study, due to differences in transcripts homology between the species [35,36,37].

In this study, we utilized a published RNAseq dataset generated from transcript sequencing of mesocarp tissues at different stages of development (WAA) as the basis to develop the probes for a custom microarray. This microarray could be used to study the gene expression profiles in the mesocarp tissue during development. The oil palm custom mesocarp microarray consists of 95,382 probes derived from the mesocarp transcripts and 1325 standard Agilent control probes, to monitor the efficiencies of the hybridization processes. In this array, each of the mesocarp transcripts was represented by three distinct probes, each 60 bp in length. The individual probes were positioned randomly on the 105K array. Three probes per transcript were used to increase confidence in outcomes by avoiding bias towards signals produced due to non-specific binding and partial degradation of particular transcripts.

**Figure 1:**
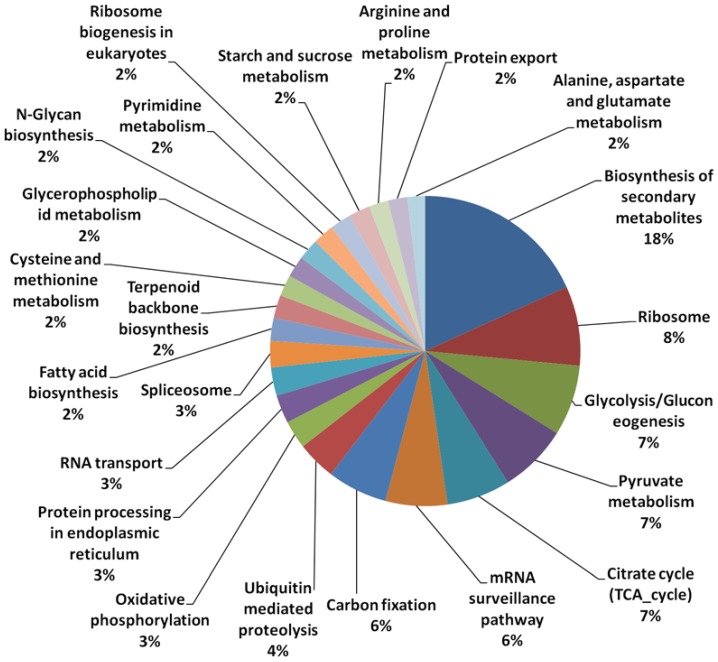
Classification of transcripts in different pathways using the Kyoto Encyclopedia of Genes and Genomes (KEGG) database.

A total of 31,804 unique contigs (assembled from approximately 3 million sequenced reads from mesocarp tissue), each denoted with an individual identifier, were used for microarray probe design. From the 31,804 contigs used, 49.3% of the transcripts were annotated based on homology to those in the Uniprot database (Table 1). A total of 8.1% of the transcripts utilized also had acceptable Kyoto Encyclopedia of Genes and Genomes (KEGG) ID matches. However, when further classified based on their putative functions in different pathways, only 1.9% could be classified uniquely into defined pathways using the KEGG database. Similar figures were observed in transcriptome sequencing of microalgae, as an example, in which only about 3.9% of the annotated sequences had GO matches and 1.7% were assigned Enzyme Commission (EC) numbers [38]. The results show the incompleteness of oil palm annotation if based solely on the KEGG database. Using the KEGG classifications however, we observed that the largest group (of 18% of the KEGG classified transcripts) were putatively involved in secondary metabolite biosynthesis (Figure 1) followed by those involved in ribosome biology (8%) glycolysis, pyruvate and citrate cycles (7% in each of the three categories). Only 2% of classified transcripts were annotated as being involved in fatty acid (FA) biosynthesis, starch and sucrose metabolism, for each class. In this custom array, unique un-annotated transcripts (>50% of the contigs, data not shown) were also included to provide better coverage of the expressed genes found in oil palm mesocarp tissue.

**Table 1:** Oil palm transcripts annotation summary.

Transcript	Number of Sequences	Percentage (%)
Transcripts selected for probe design	31,804	
Transcripts with Uniprot database hits	15,695	49.3
Transcripts with KEGG database hits	2569	8.1
Transcripts with KEGG Orthologs ID (pathways) hits	624	1.9

### 3.2. Expression Comparisons of Fatty Acid (FA), Triacyl Glyceride (TAG) Biosynthesis, Citric Acid Cycle (TCA) and Glycolysis Genes between Custom Oil Palm Mesocarp, Arabidopsis and Rice Microarrays at 16 Week After Anthesis (WAA)

**Figure 2:**
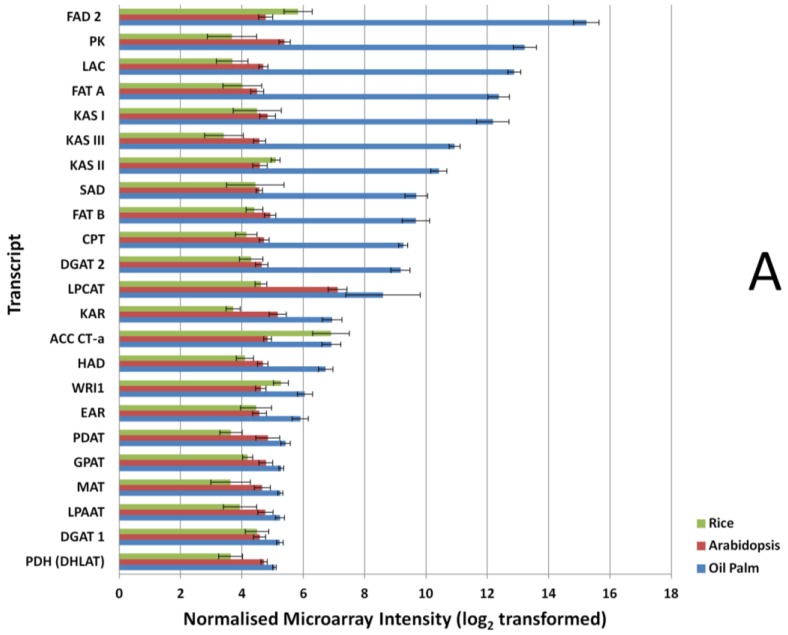

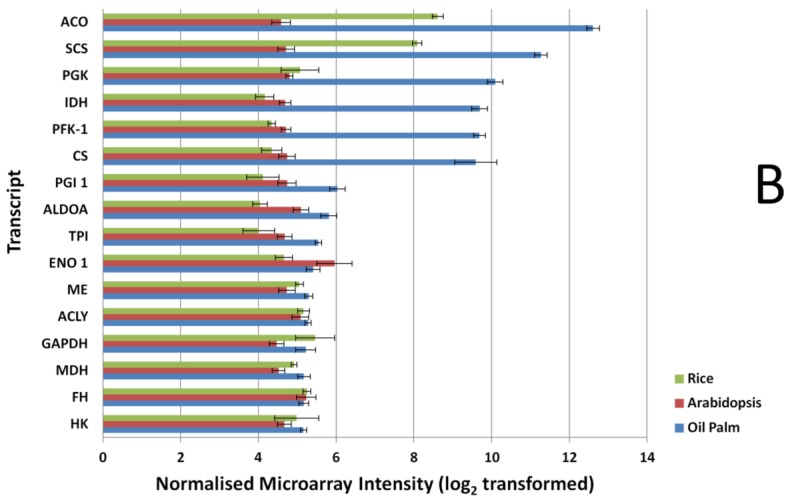
Expression signal comparison of selected fatty acid (FA) and triacylglycerols (TAGs). (**A**) tricarboxylic acid cycle (TCA) and glycolysis (**B**) genes at 16 Weeks After Anthesis (WAA) using different microarray platforms, Rice, *Arabidopsis* and Oil Palm. Signals were compared using a Mann-Whitney test at *p*-value < 0.05. FAD 2, Oleate desaturase; PK, Pyruvate kinase; LAC, Long-chain acyl-CoA synthetase; FAT A, Acyl-ACP thioesterase A; KAS I, Ketoacyl-ACP synthase I; KAS III, Ketoacyl-ACP synthase III; KAS II, Ketoacyl-ACP synthase II; SAD, Stearoyl-ACP desaturase; FAT B, Acyl-ACP thioesterase B; CPT, Diacylglycerol cholinephosphotransferase; DGAT 2, Acyl-CoA: diacylglycerol acyltransferase 2; LPCAT, 1-acyl glycerol-3-phosphocholine acyltransferase; KAR, Ketoacyl-ACP reductase; ACC CT-α, Carboxyltransferase α-subunit of acetyl-CoA carboxylase; HAD, hydroxyacyl-ACP dehydrase; WRI1, EAR, Enoyl-ACP reductase; PDAT, Phospholipid:diacylglycerol acyltransferase; GPAT, glycerol-3-phosphate acyltransferase; MAT, Malonyl-CoA:ACP malonyltransferase; LPAAT, Lyso PA acyltransferase; DGAT 1, Acyl-CoA:diacylglycerol acyltransferase 1; PDH (DHLAT), Dihydrolipoamide acetyltransferase; SCS, Succinyl coenzyme A synthetase; PGK, Phosphoglycerate kinase; IDH, Isocitrate dehydrogenase; PFK-1, Phosphofructokinase 1; CS, Citrate synthase; PGI, phosphoglucose isomerase; ALDOA, Fructose-bisphosphate aldolase; TPI, Triosephosphate isomerase; ENO 1, enolase 1; ME, Malic Enzyme; ACLY, ATP Citrate Lyase; GAPDH, Glyceraldehyde-3-phosphate dehydrogenase; MDH, Malate dehydrogenase; FH, Fumarase; HK, Hexokinase.

In the custom oil palm mesocarp microarray, probes designed to detect FA and TAG biosynthesis genes during mesocarp development were included. To compare the detection efficiencies of FA and TAG biosynthesis genes on the different microarray platforms, we compared the expression levels through signals produced from microarray hybridization in mesocarp tissue at 16 WAA, at which point mesocarp oil accumulation in this tissue is entering the exponential phase [39]. In the *Arabidopsis* and rice microarrays, the expression signals produced at 16 WAA were similar across the selected FA and TAG genes (Figure 2A), however up to 3-fold variation was observed in the oil palm microarray hybridization signals. The statistical test (Mann-Whitney test) showed that the oil palm custom array produced significantly higher (*p*-value < 0.01) probe signal intensity in all the FA and TAG probes when compared to signals produced using *Arabidopsis* microarray. Comparing the rice microarray and oil palm custom array, we also found a similar situation to the *Arabidopsis*-oil palm array comparison. The Mann-Whitney test also showed significant differences overall in the probe signal levels of transcript that code for FA and TAG genes using the oil palm microarray as compared to the rice microarray platforms, at *p*-value < 0.05. This was with the exception of the probe that coded for Carboxyltransferase α-subunit of heteromeric acetyl-CoA carboxylase (ACC CT-α), where no significant differences were seen between rice and oil palm arrays. (*p*-value, 0.66).

Similar results were also observed when comparing the glycolysis and TCA pathway genes (Figure 2B). Most of the genes exhibit higher expression signals in the oil palm microarray dataset and show significant differences in their expression (*p*-value < 0.01) when compared to the *Arabidopsis* array, with the exception of the transcript that coded for fumarase (*p*-value, 0.1689). In the *Arabidopsis* microarray, only the transcript coding for enolase 1 had significantly higher expression signal (*p*-value, 0.0004) compared to oil palm microarray. Comparisons between rice and oil palm arrays yielded similar results, with most of the genes showing significantly higher expression signal using the oil palm array, with the exception of the transcripts for hexokinase, fumarase, glyceraldehydes-3-phosphate dehydrogenase (GAPDH), and ATP citrate lyase (*p*-value > 0.05).

Probe signal comparisons identified several genes with expression signals with up to 3-fold difference between *Arabidopsis* and the oil palm microarray, similarly for the rice microarray. The exceptions were aconitase and succinyl-CoA synthetase alpha subunit; these two transcriptsexhibited much higher signal in the rice microarray compared to the Arabidopsis microarray. For genes in FA, TAG, TCA and glycolysis pathways (Figure 2), the 16 biological samples used in the microarray showed consistent differences between replicates. This argues for a lack of dynamic detection range in the *Arabidopsis* and rice microarrays, compared to the oil palm microarray even for genes in conserved metabolic pathways—A reflection of the lack of homology between the *Arabidopsis* and rice array probes used here compared with oil palm FA, TAG, glycolysis and TCA genes. 

### 3.3. FA and TAG Biosynthesis Profiling in Oil Palm Mesocarp Development (12 WAA–22 WAA) Using the Oil Palm Mesocarp Microarray

**Figure 3:**
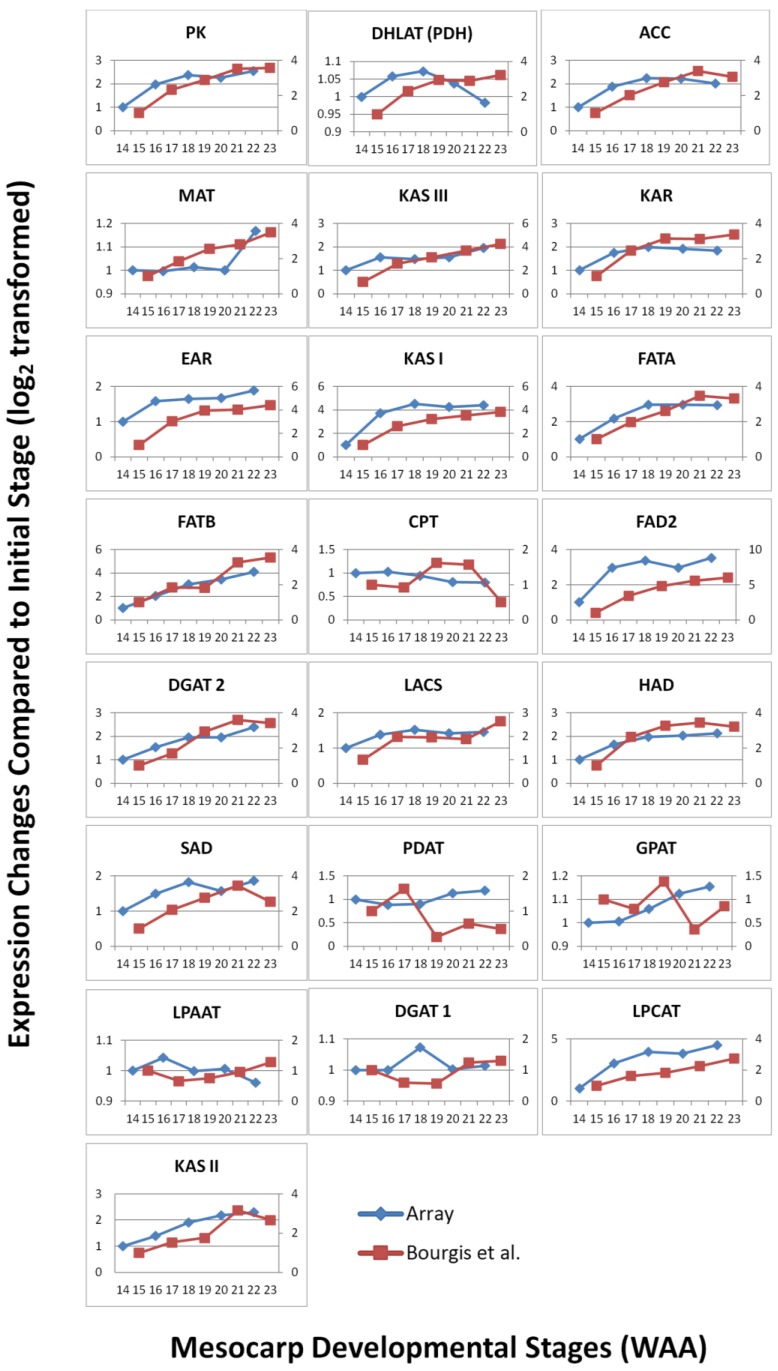
Expression change comparisons of selected FA genes between microarray and Bourgis *et al.* [4] throughout mesocarp development.

**Figure 4:**
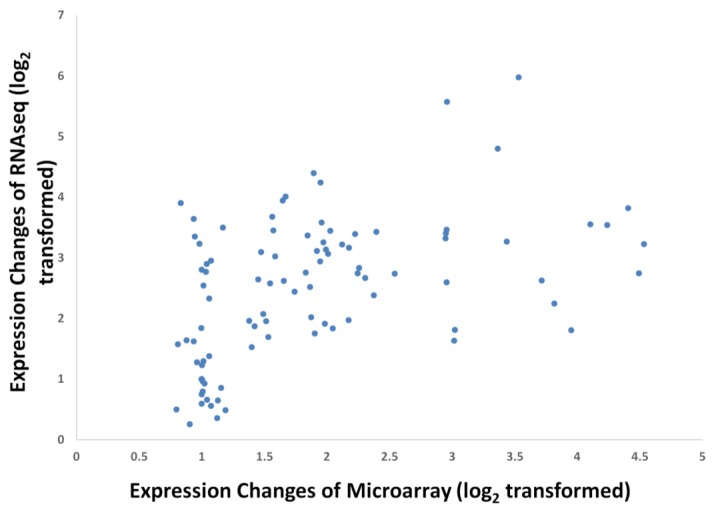
Coefficient of correlation of transcriptome sequencing [4] and microarray data for FA genes at 16 WAA. R^2^ = 0.569, *p*-value < 0.01.

To study the robustness of the oil palm mesocarp microarray, we further compared the expression profile of the selected FA and TAG biosynthesis genes throughout mesocarp development with published datasets derived from transcriptome sequencing at similar development stages. From the expression profiles of the selected genes (Figure 3), the majority of probes exhibited an increase in expression signal throughout mesocarp development. This is in concordance with Bourgis *et al.* [4] where they reported almost all the transcripts related to FA biosynthesis continue to increase until the end of oil accumulation. From our correlation study, the FA and TAGs genes show reasonable correlation (Pearson correlation) with R^2^ = 0.569 and *p*-value < 0.01 (Figure 4). Individually, 56% of these genes assessed with the custom oil palm microarray show high concordance to the published dataset, with R^2^ > 0.9 and *p*-value < 0.05 (Table 2).

**Figure 5:**
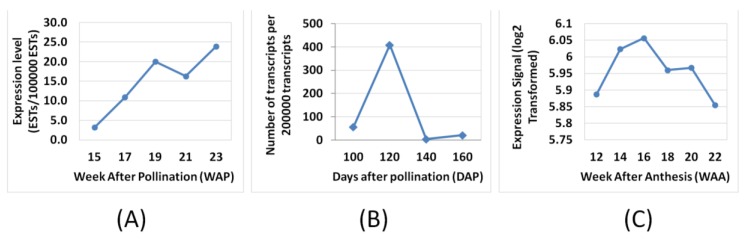
Expression comparisons of WRI1 between Bourgis *et al.* [4] (**A**), Tranbarger *et al*. [6] (**B**) and oil palm microarray (**C**).

Nonetheless, there are a few genes that exhibited differences in expression profiles derived using this microarray compared to the published data [4,6] such as LACS, SAD, MAT, PDH(DHLAT), CPT, GPAT, PDAT, DGAT 1, WRI1 and LPAAT (Figure 3). The differences could be a reflection of how the respective experiments were carried out and also differences in the technology used to assess the transcripts (hybridisation versus library sequencing). However, when we compared the WRI1 expression profile from the oil palm microarray here to Bourgis *et al*. [4] and to Tranbarger *et al*. [6] we observed similarity between these three different studies (Figure 5). We observed that the expression pattern of WRI1 in Bourgis *et al*. [4] (Figure 5A) and our microarray (Figure 5C) are similar at initial stages (12 WAA to 16 WAA) but show different trends of expression at the end of the maturation stage. However, the expression trend of WRI1 in our microarray data is similar to that reported by Tranbarger *et al*. [6] (Figure 5B) with an expression peak at 120 DAP (~17 WAA). The expression of WRI1 then declines as the mesocarp develops towards the maturation stage. 

**Table 2:** Pearson correlation of expression changes between microarray and published data [4]. * Significant at *p*-value < 0.05.

Genes	R^2^	*p*-value
HAD	0.984	0.003*
EAR	0.975	0.005*
KAR	0.963	0.008*
KAS I	0.961	0.009*
PK	0.953	0.012*
KAS III	0.941	0.017*
FATA	0.937	0.019*
DGAT2	0.927	0.023*
FATB	0.919	0.027*
ACC	0.919	0.027*
FAD2	0.913	0.03*
LPCAT	0.911	0.031*
KAS II	0.907	0.034*
LACS	0.836	0.078
SAD	0.753	0.142
MAT	0.702	0.186
DHLAT	0.209	0.736
CPT	0.018	0.978
GPAT	−0.373	0.536
PDAT	−0.438	0.461
DGAT1	−0.497	0.394
WRI1	−0.665	0.221
LPAAT	−0.884	0.047

Differences in sampling methods at different maturation stages and potentially differences in the genetic backgrounds used maycontribute to the observed differences in expression profiles of these genes. In this study, mesocarp samples of each maturation stage were harvested from all 16 selected palms, compared to the published studies where mesocarp samples of different maturation stages were harvested from different palms. We also found that the expression of specific genes can be genotype dependent (data not shown).

**Figure 6:**
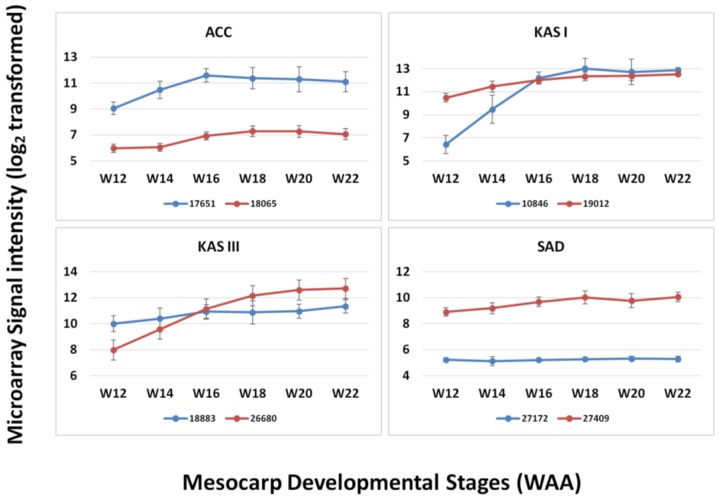
Expression of paralogs of four FA genes throughout mesocarp development.

In the design of this microarray, we also included paralog sequences from our mesocarp transcriptome sequencing that appeared in the mesocarp tissue, focusing on the genes involved in the FA biosynthesis pathway as reported in Dussert *et al*. [5]. From the comparison of paralogs of several FA genes (ACC, KAS I, KAS III and SAD), we observed that the level of expression of these paralogs is different and that these correspond to different enzymes (Figure 6). The paralogs corresponding to KAS I are different in expression level at the initial stages (12–14 WAA) but similar in their expression level from 16 WAA until maturation. In the case of KAS III, the paralog 18883 was expressed at higher levels than 26680 at the initial stages of mesocarp development (12–14 WAA) but the relative expression levels switched for these two paralogs at later stages of oil palm mesocarp development (18–22 WAA). However, in some cases paralogs showed only low levels of expression throughout mesocarp development as can be observed in the case of ACC and SAD. From the expression pattern of different paralogs (although within the same tissue, mesocarp) it can be suggested that these paralogs may play different roles in the FA biosynthesis pathway [5]. The ability to design microarray probes to detect paralogs, based on the extensive transcript sequencing potentially allows the detection of functionally different transcripts. Overall, it was observed that the expression levels of individual genes appeared more consistent using the oil palm microarray than reported using the 454 sequencing-based publications [4,6], despite the fact that 454 sequencing is known to be sensitive [40]. However, microarray experiments are based on the hybridization efficiencies of the probes and this will affect the level of signal produced. The probe efficiencies may be affected by sequence differences between transcript and probe sequence. Furthermore, the position of differences in probe sequences may also affect hybridization efficiencies and eventually signal intensity [41]. While being a disadvantage of microarray approaches compared to transcriptome sequencing, the use of siblings in this experiment combined with the 3 × 60 bp probe design should minimize the effect. The development of a custom oil palm mesocarp microarray is also justified by the increased range of response observed when genes involved in lipid biosynthesis were compared with the *Arabidopsis* microarray. While *Arabidopsis* is a highly characterized model plant with a full genome sequence that has been highly annotated, the current study suggests that it may have limited use as a cross-species microarray platform for oil palm without validation and cross-species sequence comparisons, even for metabolic pathways that are functionally conserved. 

### 3.4. Validation by Quantitative Real-time PCR (qPCR)

**Figure 7:**
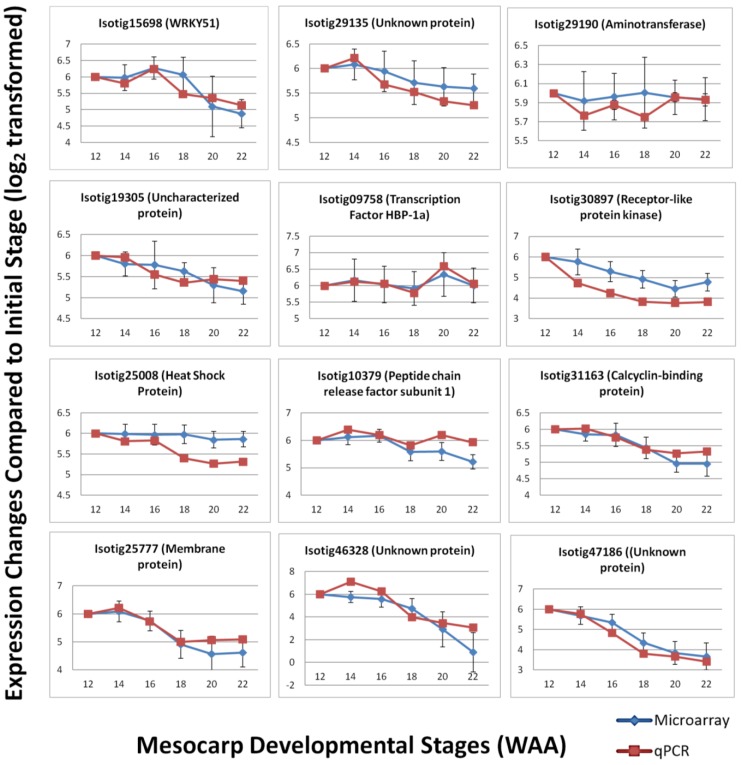
Expression trend comparison between microarray and qPCR of selected gene candidates throughout mesocarp development.

To validate the expression patterns observed using the Oil Palm mesocarp microarray platform, qPCR was performed to compare the expression level of selected candidates. qPCR serves as a validation tool due to its greater precision and better dynamic range compared to microarray [42]. A total of 12 candidates were randomly selected from the oil palm microarray experiment based on three categories of expression: high, medium and low. The selected genes included those involved in various biological pathways, including some transcripts with unknown function. Primer sets were designed based on published transcriptome sequencing data. The efficiencies of the primer sets were determined and the specificity of the primer sets were analyzed based on dissociation curves (Supplementary Data—qPCR primer efficiency). The qPCR results were normalized using in-house validated oil palm reference genes [32] and show good correlation (Pearson correlation) with the expression profiles in the microarray experiment (R^2^ = 0.729, *p*-value < 0.01). The expression profiles generally agreed with the results obtained from oil palm microarray hybridization (Figure 7) with increasing and decreasing trends over the time points in a similar pattern to the microarray expression profile over the same time points. In some cases, the signal magnitude of the microarray experiment was different from the qPCR results, with qPCR expression showing more dramatic changes between time points. This possibly reflects the greater dynamic range of detection [42,43] of qPCR and microarray platforms, which have been reported to generally underestimate the relative changes of expression level in samples tested [44]. However, the overall correlation observed between microarray and qPCR analysis indicates the utility of the oil palm custom mesocarp array to identify expression patterns and differential gene expression between samples.

## 4. Conclusions 

Due to the economic importance of oil palm (*Elaeis guineensis*), many studies have been carried out to evaluate the expression profiles of FA and TAG assembly genes in the mesocarp. Limited sequence data exists in public databases, with only recently published details on mesocarp gene expression in oil palm by Tranbarger *et al*. [6], Bourgis *et al*. [4], Dussert *et al*. [5] and Singh *et al.* [8]. In the present study, we sampled the same palms at different stages of fruit development to reduce biological variation, with all palms being sibling or half-siblings. By developing a mesocarp oil palm microarray, we demonstrate an alternative to the study of gene expression profile in oil palm mesocarp through sequencing. 

This study reports the first development of a 105K (3 × 60 bp probe per isotig) oligonucleotide microarray for oil palm mesocarp gene expression studies. This microarray is an important tool to capture overall gene expression changes in oil palm mesocarp tissue throughout its developmental and maturation stages. The oil palm mesocarp gene expression array was found to be far superior to the Agilent *Arabidopsis* and rice gene expression array, in terms of signal produced and dynamic signal range. The signals produced using the same RNA samples were generally greater with the probes of the oil palm mesocarp microarray compared to probes of *Arabidopsis* and rice microarray, as would be expected, despite a likely functional conservation of genes involved in oil biosynthesis between species. Our qPCR validation of the results from the oil palm microarray show good correlation with most of the cases studied and this adds weight to the robustness of this microarray in future oil palm research. Within oil palm expression, the trends of targeted FA genes that were revealed using the microarray show reasonable similarity when compared to published, transcriptome sequencing studies, although some differences were noted. We believe the use of this oil palm mesocarp microarray provides opportunities to further understand the biological changes in the mesocarp tissue during development, especially transcriptional changes during oil development in different germplasm. It can also be used to identify the key regulators and genes that drive the genetic improvement of yield in oil palm. The lower cost of the array approach potentially allows larger populations with more biological replicates to be analyzed and, given the highly heterozygous and heterogeneous nature of oil palm, this is an important consideration.

## Supplementary Files

Supplementary File 1

## References

[1] Tan K.T., Lee K.T., Mohamed A.R., Bhatia S. (2009). Palm oil: Addressing issues and towards sustainable development. Renew. Sust. Energ. Rev..

[2] Montoya C., Lopes R., Flori A., Cros D., Cuellar T., Summo M., Espeout S., Rivallan R., Risterucci A.-M., Bittencourt D. (2013). Quantitative trait loci (QTLs) analysis of palm oil fatty acid composition in an interspecific pseudo-backcross from *Elaeis oleifera* (H.B.K.) Cortés and oil palm (*Elaeis guineensis* Jacq.). Tree Genet. Genomes.

[3] Voelker T. (2011). Secrets of palm oil biosynthesis revealed. Proc. Natl. Acad. Sci. USA.

[4] Bourgis F., Kilaru A., Cao X., Ngando-Ebongue G.F., Drira N., Ohlrogge J.B., Arondel V. (2011). Comparative transcriptome and metabolite analysis of oil palm and date palm mesocarp that differ dramatically in carbon partitioning. Proc. Natl. Acad. Sci. USA.

[5] Dussert S., Guerin C., Andersson M., Joët T., Tranbarger T.J., Pizot M., Sarah G., Omore A., Durand-Gasselin T., Morcillo F. (2013). Comparative transcriptome analysis of three oil palm fruit and seed tissues that differ in oil content and fatty acid composition. Plant Physiol..

[6] Tranbarger T.J., Dussert S., Joet T., Argout X., Summo M., Champion A., Cros D., Omore A., Nouy B., Morcillo F. (2011). Regulatory mechanisms underlying oil palm fruit mesocarp maturation, ripening, and functional specialization in lipid and carotenoid metabolism. Plant Physiol..

[7] Singh R., Low E.-T.L., Ooi L.C.-L., Ong-Abdullah M., Ting N.-C., Nagappan J., Nookiah R., Amiruddin M.D., Rosli R., Manaf M.A.A. (2013). The oil palm shell gene controls oil yield and encodes a homologue of seedstick. Nature.

[8] Singh R., Ong-Abdullah M., Low E.-T.L., Manaf M.A.A., Rosli R., Nookiah R., Ooi L.C.-L., Ooi S.-E., Chan K.-L., Halim M.A. (2013). Oil palm genome sequence reveals divergence of interfertile species in old and new worlds. Nature.

[9] Lorenz W.W., Alba R., Yu Y.-S., Bordeaux J., Simoes M., Dean J. (2011). Microarray analysis and scale-free gene networks identify candidate regulators in drought-stressed roots of loblolly pine (*P. taeda* L.). BMC Genomics.

[10] Jain R., Dey B., Tyagi A. (2012). Development of the first oligonucleotide microarray for global gene expression profiling in guinea pigs: Defining the transcription signature of infectious diseases. BMC Genomics.

[11] Gardner L., Jayasundara N., Castilho P., Block B. (2012). Microarray gene expression profiles from mature gonad tissues of Atlantic bluefin tuna, *Thunnus thynnus* in the gulf of mexico. BMC Genomics.

[12] Wang Y., Ma H., Liu G., Xu C., Zhang D., Ban Q. (2008). Analysis of gene expression profile of *Limonium bicolor* under NaHCO_3_ stress using cDNA microarray. Plant Mol. Biol. Rep..

[13] Lee Y.-P., Yu G.-H., Seo Y., Han S., Choi Y.-O., Kim D., Mok I.-G., Kim W., Sung S.-K. (2007). Microarray analysis of apple gene expression engaged in early fruit development. Plant Cell Rep..

[14] Kathiresan A., Lafitte H.R., Chen J., Mansueto L., Bruskiewich R., Bennett J. (2006). Gene expression microarrays and their application in drought stress research. Field Crops Res..

[15] Payton P., Kottapalli K., Rowland D., Faircloth W., Guo B., Burow M., Puppala N., Gallo M. (2009). Gene expression profiling in peanut using high density oligonucleotide microarrays. BMC Genomics.

[16] Seki M., Narusaka M., Ishida J., Nanjo T., Fujita M., Oono Y., Kamiya A., Nakajima M., Enju A., Sakurai T. (2002). Monitoring the expression profiles of 7000 Arabidopsis genes under drought, cold and high-salinity stresses using a full-length cDNA microarray. Plant J..

[17] Bagnaresi P., Moschella A., Beretta O., Vitulli F., Ranalli P., Perata P. (2008). Heterologous microarray experiments allow the identification of the early events associated with potato tuber cold sweetening. BMC Genomics.

[18] Moore S., Payton P., Wright M., Tanksley S., Giovannoni J. (2005). Utilization of tomato microarrays for comparative gene expression analysis in the Solanaceae. J. Exp. Bot..

[19] Teh H.F., Neoh B.K., Hong M.P.L., Low J.Y.S., Ng T.L.M., Ithnin N., Thang Y.M., Mohamed M., Chew F.T., Yusof H.M. (2013). Differential metabolite profiles during fruit development in high-yielding oil palm mesocarp. PLoS One.

[20] Neoh B.K., Teh H.F., Ng T.L.M., Tiong S.H., Thang Y.M., Ersad M.A., Mohamed M., Chew F.T., Kulaveerasingam H., Appleton D.R. (2013). Profiling of metabolites in oil palm mesocarp at different stages of oil biosynthesis. J. Agric. Food Chem..

[21] Conesa A., Götz S., García-Gómez J.M., Terol J., Talón M., Robles M. (2005). Blast2GO: A universal tool for annotation, visualization and analysis in functional genomics research. Bioinformatics.

[22] Kanehisa M., Goto S. (2000). KEGG: Kyoto encyclopedia of genes and genomes. Nucleic Acids Res..

[23] Uniprot Database. http://www.uniprot.org/.

[24] eArray. https://earray.chem.agilent.com/earray/.

[25] Model Organism Gene Expression Microarrays—Details & Specifications. http://www.genomics.agilent.com/CollectionSubpage.aspx?PageType=Product&SubPageType=ProductData&PageID=1508.

[26] Feature Extraction Software. http://www.genomics.agilent.com/en/product.jsp?cid=AG-PT-144&tabId=AG-PR-1050&_requestid=336047.

[27] Ritchie M.E., Diyagama D., Neilson J., van Laar R., Dobrovic A., Holloway A., Smyth G. (2006). Empirical array quality weights in the analysis of microarray data. BMC Bioinformatics.

[28] Ritchie M.E., Silver J., Oshlack A., Holmes M., Diyagama D., Holloway A., Smyth G.K. (2007). A comparison of background correction methods for two-colour microarrays. Bioinformatics.

[29] Smyth G., Gentleman R., Carey V.J., Huber W., Irizarry R.A., Dudoit S. (2005). Limma: Linear models for microarray data. Bioinformatics and Computational Biology Solutions Using R and Bioconductor.

[30] Primer Premier. http://www.premierbiosoft.com/primerdesign/index.html.

[31] Hellemans J., Mortier G., de Paepe A., Speleman F., Vandesompele J. (2007). qBase relative quantification framework and software for management and automated analysis of real-time quantitative PCR data. Genome Biol..

[32] Yeap W.-C., Loo J., Wong Y., Kulaveerasingam H. (2013). Evaluation of suitable reference genes for qRT-PCR gene expression normalization in reproductive, vegetative tissues and during fruit development in oil palm. Plant Cell Tiss. Organ Cult..

[33] Vandesompele J., de Preter K., Pattyn F., Poppe B., van Roy N., de Paepe A., Speleman F. (2002). Accurate normalization of real-time quantitative RT-PCR data by geometric averaging of multiple internal control genes. Genome Biol..

[34] Davey M., Graham N., Vanholme B., Swennen R., May S., Keulemans J. (2009). Heterologous oligonucleotide microarrays for transcriptomics in a non-model species; a proof-of-concept study of drought stress in Musa. BMC Genomics.

[35] Ji W., Zhou W., Gregg K., Yu N., Davis S. (2004). A method for cross-species gene expression analysis with high-density oligonucleotide arrays. Nucleic Acids Res..

[36] Grigoryev D., Ma S.-F., Simon B., Irizarry R., Ye S., Garcia J. (2005). *In vitro* identification and *in silico* utilization of interspecies sequence similarities using genechip® technology. BMC Genomics.

[37] Chismar J.D., Mondala T., Fox H.S., Roberts E., Langford D., Masliah E., Salomon D.R., Head S.R. (2002). Analysis of result variability from high-density oligonucleotide arrays comparing same-species and cross-species hybridizations. BioTechniques.

[38] Rismani-Yazdi H., Haznedaroglu B., Bibby K., Peccia J. (2011). Transcriptome sequencing and annotation of the microalgae dunaliella tertiolecta: Pathway description and gene discovery for production of next-generation biofuels. BMC Genomics.

[39] Nair K.P.P., Nair K.P.P. (2010). Oil palm (Elaeis guineensis Jacquin). The Agronomy and Economy of Important Tree Crops of the Developing World.

[40] Sîrbu A., Kerr G., Crane M., Ruskin H.J. (2012). RNA-Seq *vs.* dual- and single-channel microarray data: Sensitivity analysis for differential expression and clustering. PLoS One.

[41] Jakubek Y., Cutler D. (2012). A model of binding on DNA microarrays: Understanding the combined effect of probe synthesis failure, cross-hybridization, DNA fragmentation and other experimental details of affymetrix arrays. BMC Genomics.

[42] Allanach K., Mengel M., Einecke G., Sis B., Hidalgo L.G., Mueller T., Halloran P.F. (2008). Comparing microarray *versus* RT-PCR assessment of renal allograft biopsies: Similar performance despite different dynamic ranges. Am. J. Transplant..

[43] Draghici S., Khatri P., Eklund A.C., Szallasi Z. (2006). Reliability and reproducibility issues in DNA microarray measurements. Trends Genet..

[44] Yuen T., Wurmbach E., Pfeffer R.L., Ebersole B.J., Sealfon S.C. (2002). Accuracy and calibration of commercial oligonucleotide and custom cDNA microarrays. Nucleic Acids Res..

